# The complete chloroplast genome of *Viola philippica* (Violaceae)

**DOI:** 10.1080/23802359.2021.1894998

**Published:** 2021-03-24

**Authors:** Yuqi Yi, Pingping Lin, Jiaqi Ruan, Emelda Rosseleena Rohani, Rongchun Han

**Affiliations:** a School of Pharmacy, Anhui University of Chinese Medicine, Hefei, China; b Institute of Systems Biology (INBIOSIS), Universiti Kebangsaan Malaysia, Bangi, Malaysia

**Keywords:** Chloroplast genome, Violaceae, *Viola philippica*

## Abstract

*Viola philippica*, as a traditional Chinese medicine, has great value in treating various diseases. Here, we report the chloroplast genome of *V. philippica* and its phylogenetic feature. The complete chloroplast genome is 156,744 bp in length, assembled from 22,346,570 reads, and its GC contents ratio is 36.26%. Its long single-copy (LSC) region is 85,892 bp. The small single-copy (SSC) region covers 18,006 bp and inverted repeat (IR) is 26,423 bp. It encodes 77 genes, including 43 protein genes, 4 rRNA genes, and 30 tRNA genes. Moreover, according to the phylogenetic analysis for a total of 12 chloroplast sequences, *V. philippica* demonstrated close relationship within genus *Viola*.

*Viola philippica* is a traditional Chinese medicine, which has been used for more than 2000 years (Zhang et al. 2012). In ancient China, it was used for detoxification and reducing swelling (Pan et al. 2015). Because of its strong adaptability to different ecological environments, the plant thrives in China (Huang et al. 2009). Today, it is also widely prescribed in treating inflammatory diseases (Jeong et al. 2016) and bacterial infection diseases (Sun et al. 2011). Studies on its functional genes and related expression analysis have been undertaken (Jian et al. 2015), but information on the complete chloroplast genome is still insufficient. In this study, we assembled and analyzed *V. philippica* chloroplast genome in order to provide new insights for future research on this medicinal plant.

Fresh leaves of *V. philippica* were collected from the botanical garden in Anhui University of Chinese Medicine (N31°56′29ʺ; E117°23′21ʺ). We deposited the specimen in Herbarium of Anhui University of Chinese Medicine under the voucher number 200713AH008. CTAB method was used to extract total genomic DNA (Healey et al. 2014). After DNA quality check and concentration quantification, the following high-throughput sequencing was conducted by Genewiz Co. Ltd. (Suzhou, China). The resultant raw data were subjected to quality control and assembled by Velvet software (Zerbino and Birney 2008). Then, the contigs were gapfilled by GapFiller (Boetzer et al. 2011). To predict gene information from the chloroplast genome, Prodigal (V2.6.3) was adopted and gene annotation was fulfilled by Diamond (V0.8.15) against NR database and Blast (V2.2.28+) against KEGG . Finally, chloroplast genomic features for *V. philippica* were submitted to GenBank with the accession number MW229048.

The chloroplast genome was 100% covered by clean reads and it was found that whole length of the genome is 156,744 bp, resulted from 22,346,570 reads (3,334,879,247 bases) with GCs ratio as 36.26%. The genome contains 77 genes in total, including 43 protein-encoding genes, 4 rRNAs and 30 tRNAs. In addition, its long single-copy (LSC) region is 85,892 bp and the small single-copy region is 18,006 bp, with inverted repeat (IR) as 26,423 bp.

*Viola philippica* chloroplast genome was previously reported with the accession number MT796627 in GenBank database. We compared MT796627 and MW229048 in terms of nucleotide composition and sequence accuracy. Supplementary Table 1 illustrated all 201 single nucleotide polymorphisms. Regarding oligo nucleotide differences between the two sequences, 61 locations were identified (Supplementary Table 2). In order to assess sequence accuracy, we designed four pairs of primers (Supplementary Table 3) for PCR verification and found that MW229048 was more reliable (Supplementary Figure 1).

To evaluate the genetic relationship between *V. philippica* and relevant species, we retrieved 12 chloroplast sequences based on plant taxonomy to construct a phylogenetic tree. MEGA X was applied to conclude alignment as well as a maximum likelihood (ML) phylogenetic tree, with the combined bootstrap method (1000 replicates). For ML analysis, we also used the Tamura-Nei substitution model . In line with our expectations, *V. philippica* [MW229048] showed closer relationship with those from the same genus, compared with species from other taxonomic groups (Figure 1).

**Figure 1.**
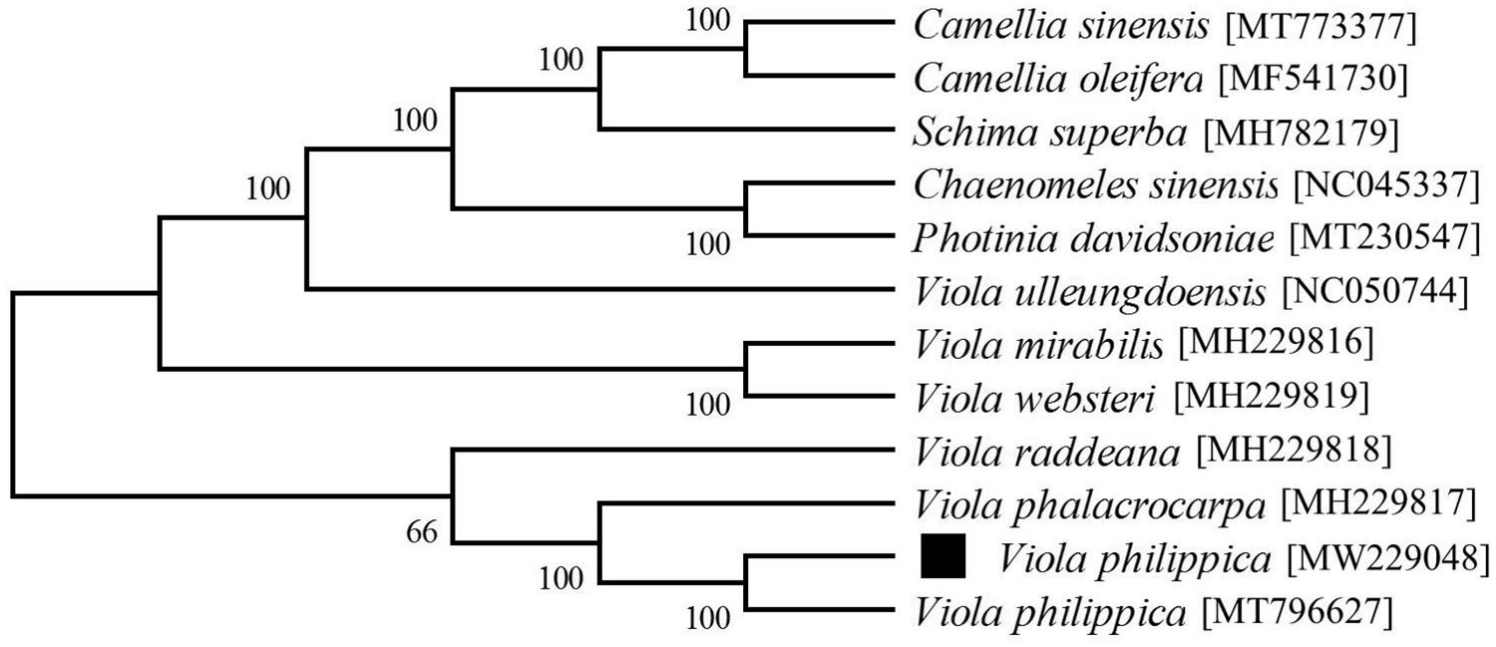
Maximum likelihood phylogenetic tree resulted from the chosen genome information of 12 relevant chloroplast sequences. Values next to the branches stand for the percentage of replicate trees where the groups clustered together.

## Data Availability

The genome sequence data that support the findings of this study are openly available in GenBank of NCBI at https://www.ncbi.nlm.nih.gov under the accession number MW229048. The associated BioProject, SRA, and Bio-Sample numbers are PRJNA689919, SRR13364241, and SAMN17221883 respectively.
